# Phylogenetic, Expression, and Bioinformatic Analysis of the *ABC1* Gene Family in *Populus trichocarpa*

**DOI:** 10.1155/2013/785070

**Published:** 2013-09-15

**Authors:** Zhanchao Wang, Haizhen Zhang, Jingli Yang, Yunlin Chen, Xuemei Xu, Xuliang Mao, Chenghao Li

**Affiliations:** ^1^State Key Laboratory of Forest Genetics and Tree Breeding, Northeast Forestry University, 26 Hexing Road, Harbin 150040, China; ^2^Library of Northeast Forestry University, 26 Hexing Road, Harbin 150040, China

## Abstract

We studied 17 *ABC1* genes in *Populus trichocarpa*, all of which contained an *ABC1* domain consisting of about 120 amino acid residues. Most of the *ABC1* gene products were located in the mitochondria or chloroplasts. All had a conserved VAVK-like motif and a DFG motif. Phylogenetic analysis grouped the genes into three subgroups. In addition, the chromosomal locations of the genes on the 19 *Populus* chromosomes were determined. Gene structure was studied through exon/intron organization and the MEME motif finder, while heatmap was used to study the expression diversity using EST libraries. According to the heatmap, *PtrABC1P14* was highlighted because of the high expression in tension wood which related to secondary cell wall formation and cellulose synthesis, thus making a contribution to follow-up experiment in wood formation. Promoter *cis*-element analysis indicated that almost all of the *ABC1* genes contained one or two *cis*-elements related to ABA signal transduction pathway and drought stress. Quantitative real-time PCR was carried out to evaluate the expression of all of the genes under abiotic stress conditions (ABA, CdCl_2_, high temperature, high salinity, and drought); the results showed that some of the genes were affected by these stresses and confirmed the results of promoter *cis*-element analysis.

## 1. Introduction 

The protein kinase-like (PKL) superfamily, which can be divided into eukaryotic protein kinases (ePKs) and atypical protein kinases (aPKs), displays enormous variability in structure and sequence [1]. The ABC1 protein family is found widely in eukaryotes and prokaryotes and has been described as a new kind of putative kinase [2]. Before *ABC1* genes were found in plants, they were identified in yeast, the first of which was named *ABC1/COQ8 *(hereafter called* ScCOQ8*). ScCOQ8 is a nuclear-encoded protein required for ubiquinone (UQ) synthesis in the mitochondria. This gene was found to be necessary for redox activity of the mitochondrial bc1 complex involved in cellular respiration, and was thus given the name *abc1* because of its activity [3]. Loss of the *ScCOQ8 *gene causes a UQ deficiency and accumulation of the biosynthetic precursor 3-hexaprenyl-4-hydroxybenzoic acid, leading to instability of the bc1 complex and a lack of bc1 activity [4]. Missteps in the original analysis of *ScCOQ8* gene function have caused confusion. The *ScCOQ8 *gene was initially believed to suppress a deleterious mutation in a cytochrome *b* translational activator (cbs2-223), leading to the incorrect conclusion that ScCOQ8p functions as a chaperone of cytochrome *b* [3, 5]. Not until a decade later was it found that *ABC1* was the *ScCOQ8 *gene and that suppression of the translational activator mutant (cbs2-223) was due to a neighboring tRNA^Trp^ gene [4, 6].

Many studies have shown the localization of the *ABC1* gene products in* Arabidopsis*, rice, and maize. Analysis of purified plastoglobules (PGs) from *Arabidopsis* chloroplasts identified six ABC1 kinase proteins (AtABC1P1, 3, 7) that localize predominantly to the plastid [7–9]. A seventh protein (AtABC1P8/OSA1) was shown to localize to the inner plastid envelope [10]. Furthermore, *ABC1P13 *is expected to localize to the mitochondria, based on the localization of its functional homolog in Baker's yeast (*Saccharomyces cerevisiae*) [11, 12]. Proteomic analysis of maize leaf fractions further supported the plastid localization of eight ABC1 kinase proteins, all of which were identified in maize proplastids, chloroplast fractions, or plastid nucleoids [13].

Jasinski et al. showed that the ABC1 proteins have the most conserved kinase motifs, including the VAIK catalytic motif (VAVK and VAMK) and the VAVK motif exists in the ABC1 domain, which also contains another motif, DFG [11, 14]. Many studies have also examined the ABC1 kinase protein domain. According to Lundquist et al. [15], a common ABC1 kinase domain is observed spanning approximately 350 residues and containing 12 conserved motifs based on an alignment of 100 full-length ABC1 kinase protein sequences from seven angiosperm species. Furthermore, eight of the ten key residues of the PKL superfamily identified in their study are present in the ABC1 kinase family and correspond to motifs III, IVa, IVb, VIIb, and VIII, which are involved in ATP binding and orientation (III, IVa, and IVb), catalysis (VIIb), and Mg^2+^ chelation (VIII) [2, 16–18]. The other seven motifs of the ABC1 kinase domain (I, II, V, VI, VIIa, IX, and X) do not have homologous sequences in ePKs, but can be found in several proteins outside of the PKL superfamily that have diverse enzyme activities.

 A recent survey in *Arabidopsis *suggested that the chloroplast AtOSA1 protein, a homolog of the ABC1 proteins, was a new factor that plays a role in the balance of oxidative stress [11, 12]. There has also been research showing that ABC1 proteins are related to abiotic stress tolerance. Wang et al. showed that *TaABC1*, a member of the ABC1 protein kinase family in wheat, could enhance tolerance to drought, salt, and cold stress when overexpressed in *Arabidopsis* [19]. While research on the ABC1 protein family has been reported in *Arabidopsis *and rice, it has not been studied in *Populus.*

In this study, we identified 17 *ABC1* genes in *Populus trichocarpa *and carried out phylogenetic and bioinformatic analysis using a number of databases and websites. We then constructed a chromosomal localization diagram using drawing tools based on transcription direction and plotting scale. Finally, we examined the relative expression levels of all 17 genes in response to various abiotic stresses by qRT-PCR using a four-step program to remove primer dimers. Our results showed the phylogenetic relationships and chromosomal locations of the *ABC1* genes in *P. trichocarpa. *Nearly all of the genes contained some *cis*-elements involved in ABA and drought stress responses, which corresponded with the results of qRT-PCR analysis. Some of the genes had high expression levels in response to stress and might be important for subsequent studies.

## 2. Materials and Methods

### 2.1. Identification of *ABC1* Genes

The Pfam (http://pfam.sanger.ac.uk/), Phytozome (http://www.phytozome.net/search.php), and NCBI (http://www.ncbi.nlm.nih.gov/) databases were searched for *ABC1* protein and CDSs. The SMART database (http://smart.embl-heidelberg.de/) was used to analyze the ABC1 domain. WoLF PSORT (http://wolfpsort.org/) was used to predict the localization of the *ABC1P* gene products [22].

### 2.2. Phylogenetic Tree

Multiple sequence alignment was done using the ClustalX 1.83 software [23] and adjusted manually with BioEdit [24]. A phylogenetic tree was constructed using the neighbor-joining method with bootstrapping analysis in MEGA5 [25]. Due to the distant relationship between *Populus *and rice, maximum parsimony (MP) method was not suitable [26]. Since maximum likelihood (ML) method was tedious, it was also not an appropriate method. The neighbor-joining method was suitable for distant relationship plants and had a simple algorithm. Taken together, NJ method was a preferred tool to construct a phylogenetic tree. Rice protein sequences were obtained from the rice annotation database (http://rice.plantbiology.msu.edu/) based on the results of Yang et al. [14].

### 2.3. Chromosomal Localization

The chromosomal localization of all of the genes was determined with PopGenIE (http://www.popgenie.org/). A diagram of their locations on the 19 chromosomes was created with drawing tools on the basis of transcription direction and plotting scale.

### 2.4. Exon Intron Structure and Motif Analysis

Exon/intron structure was investigated using the Gene Structure Display Server (CSDS) (http://gsds.cbi.pku.edu.cn/chinese.php). The coding and genomic sequences were obtained from the NCBI database (http://www.ncbi.nlm.nih.gov/). Conserved motifs for each* ABC1* gene were investigated using the Multiple Expectation Maximization for Motif Elucidation (MEME) system (Version 3.0 http://meme.sdsc.edu/meme/cgi-bin/meme.cgi).

### 2.5. Heatmap Analysis

To study the expression differences among different tissues in *Populus*, heatmap analysis was carried out using EST libraries representing gene models within PopulusDB [27, 28], which included 17 libraries that were derived from several taxa of *Populus*; aspen (*Populus tremula*), a hybrid aspen (*P. tremula *×* tremuloides* T89), and black cottonwood (http://popgenie.org/book/digital-northern).

### 2.6. Promoter Cis-Elements Identification and Analysis

Promoter sequences, the 2000 bp sequence upstream of the translational start site, of the genes were found in NCBI and analyzed in the Plant Care database (http://bioinformatics.psb.ugent.be/webtools/plantcare/ html/) which listed all of the *cis*-elements of these genes.

### 2.7. Plant Materials Treatment

Leaves of *P. trichocarpa *were treated with five abiotic stresses including ABA, CdCl_2_, NaCl, drought and high temperature. For ABA, CdCl_2_, and NaCl treatment, the leaves were steeped in 100 *μ*M ABA, 10 mM CdCl_2_, or 200 *μ*M NaCl. For drought treatment, the leaves were steeped in 4% PEG6000. All of these treatments were carried out for 4 or 8 h. For high temperature treatment, the leaves were incubated at 42°C for 0.5 or 1 h. Harvested leaves were frozen in liquid nitrogen immediately and stored at −80°C until RNA isolation.

### 2.8. RNA Isolation and Reverse Transcription

Total RNA was extracted from leaves that were treated under both control and stress conditions using the CTAB method. RNA integrity was verified by 2% agar gel electrophoresis. First-strand cDNA was synthesized using ReverTra Ace qPCR RT Master Mix with a gDNA Remover Kit (TOYOBO, Osaka, Japan), which eliminated the need for a DNA removal step. RNA samples were incubated at 65°C for 5 min. Then, 2 *μ*L of 4× DN Master Mix (gDNA Remover) was added, and ddH_2_O was used to make the volume up to 8 *μ*L. After incubation at 37°C for 5 min, 2 *μ*L of 5× RT Master Mix II was added to give a total volume of 10 *μ*L. Finally, the samples were incubated at 37°C for 15 min and 98°C for 5 min to obtain the first-strand cDNA. The reverse transcription products were used to carry out real-time quantitative PCR. Gene-specific primers for all 17 *ABC1* genes were designed based on the CDSs using Primer Premier 5 with the following parameters: melting temperature 60–63°C, primer length 20–25 bp, and PCR product length 80–180 bp. All primer sequences are shown in Table 4. 

### 2.9. Real-Time Quantitative PCR

qRT-PCR was carried out using SYBR Premix Ex Taq II (TaKaRa, Dalian, China). Reactions were prepared in a total volume of 20 *μ*L containing 10 *μ*L of 2× SYBR Premix, 6 *μ*L of ddH_2_O, 2 *μ*L cDNA template, and 2 *μ*L of F + R primers whose concentrations were 10 *μ*M. Before the final experiment, a pretest was carried out to determine the optimal reaction condition. Usually, two-step method was applied to qRT-PCR. However, this method might produce more primer dimers. To solve this problem, we tried a four-step program and got the most precise results. The reactions were performed under the following conditions: an initial denaturation step of 95°C for 1 min, followed by a three-step thermal cycling profile of denaturation at 95°C for 5 s, primer annealing at 55°C for 30 s, and extension at 72°C for 30 s. After that, an additional step of 80°C for 1 s was carried out to remove primer dimers, followed by plate reading. These procedures were conducted for 45 cycles. To verify the specificity of the amplicon for each primer pair, a melting curve analysis was performed ranging from 55°C to 99°C with a temperature increase rate of 0.2°C/s, held for 1 s. The relative quantification method (2^−ΔΔCT^) was used to evaluate quantitative variation between replicates [29]. Three technical replicates were performed for each sample to ensure the accuracy of the results.

## 3. Results

**Figure 1:**
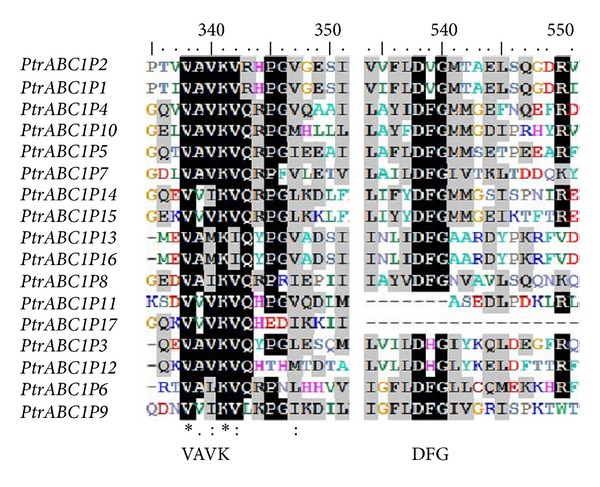
Conserved amino acid conservation sequences in the ABC1 domain of the *ABC1P* gene family in *P. trichocarpa*. The multiple alignment of the ABC1 domain was obtained with Clustal X. Fully and partially conserved (present in more than 50% of aligned sequences) residues are highlighted in black and gray, respectively. VAVK and DFG motifs are marked.

According to the Pfam, Phytozome, and NCBI databases, we identified 17 *ABC1* genes in *Populus,* which are shown in Table 1. WoLF PSORT was used to predict the localization of their gene products in the plant cell. Most of the *ABC1* gene products were located in the mitochondria or chloroplasts. The SMART database (http://smart.embl-heidelberg.de) was used to search the domains of these genes and showed that all 17 *ABC1* genes had an ABC1 domain that contained about 120 amino acid residues with a highly conserved core region. In addition, the ABC1 domain contained a VAVK-like motif (such as VAVK, VVIK, VAMK, or VVVK) and a DFG-like motif (such as DFG, DHG, or DVG) (Figure 1). Interestingly, these results coincided with those of Yang et al. [14].

**Figure 2:**
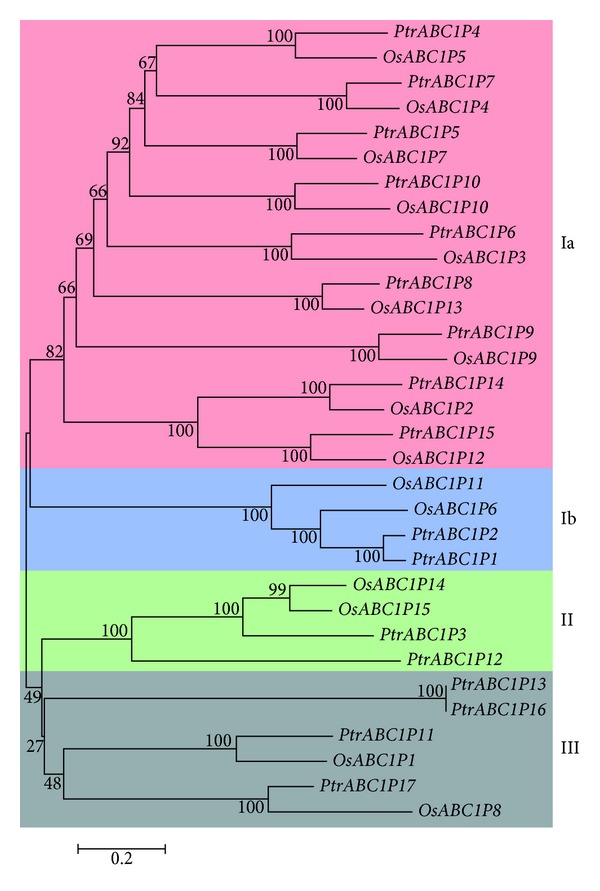
Phylogenetic tree of the ABC1 protein families in *P. trichocarpa* and rice. The tree was generated using the MEGA5 program with the neighbor-joining method. Pink represents group Ia, blue represents group Ib, green represents group II, and gray represents group III. OsABC1P represents ABC1 proteins in rice, while *PtrABC1P* represents ABC1 proteins in *P. trichocarpa. *

To evaluate the evolutionary relationships of the* ABC1* gene family, we constructed a phylogenetic tree from *P. trichocarpa *and rice protein sequences using MEGA5, which was divided into three groups (groups I, II, and III) (Figure 2) in accordance with the results of Yang et al. [14]. In their study, the rice *ABC1* genes were also divided into three groups on the basis of orthologs or paralogs, which indicated that most *ABC1P* genes in this family were not formed by duplication after the divergence of *Arabidopsis* and rice and might have expanded in a non-species-specific manner.

**Figure 3:**
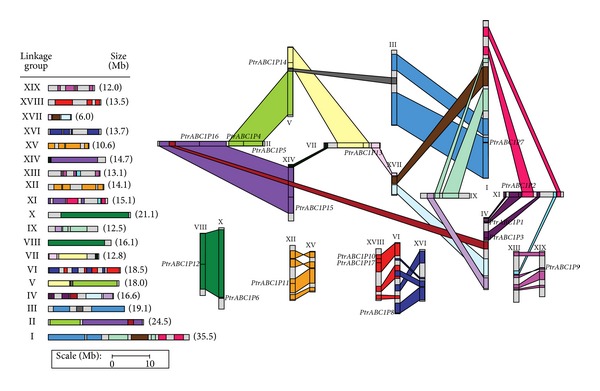
Genomic distribution of *ABC1* genes on the *P. trichocarpa* chromosomes. A schematic view of chromosome reorganization by recent whole-genome duplication in *Populus* is shown (adapted from [20]). Regions that are assumed to correspond to homologous genome blocks are shaded in the same color and connected with lines.

Chromosomal localization was determined using PopGenIE (http://www.popgenie.org/), and we created a diagram of the results using drawing tools according to transcription direction and plotting scale. This is shown in Figure 3. We found that no *ABC1* genes were located on chromosomes 3, 6, 9, 13, 15, 16, or 17. Chromosome 2 had the largest number of *PtrABC1* genes (three) followed by chromosomes 4 and 18 (two each). Only one *PtrABC1* gene was found on chromosomes 1, 5, 6, 7, 8, 10, 11, 12, 14, and 19. No substantial clustering of *Populus ABC1* genes was observed.

**Figure 4:**
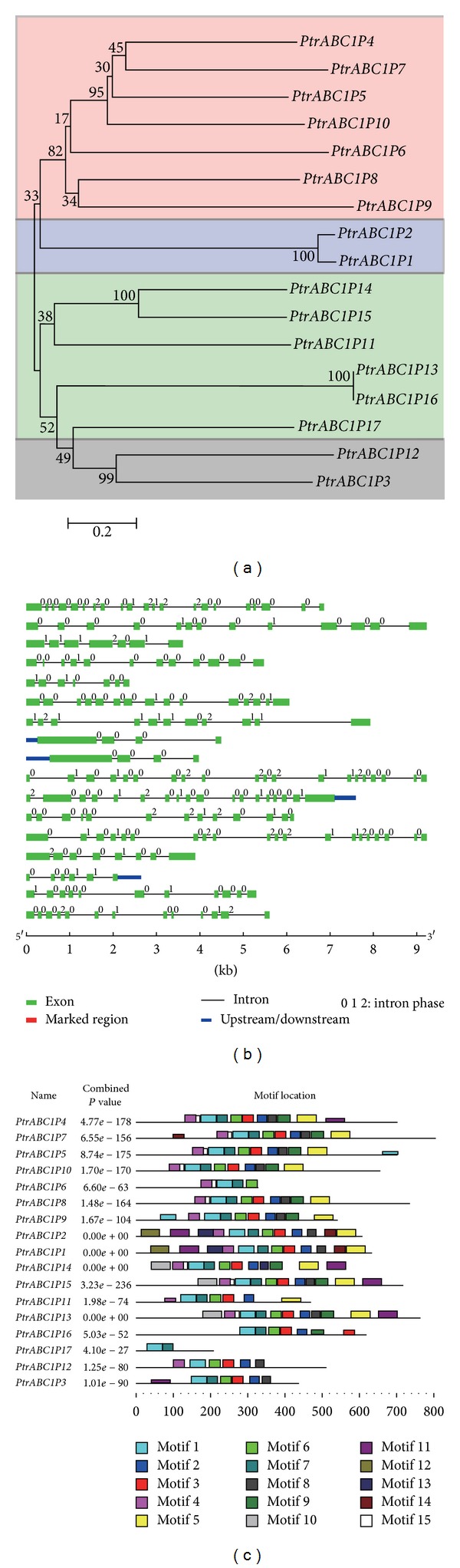
Phylogenetic analysis of the 17 *P. trichocarpa ABC1* genes (a) with the integration of exon/intron structures (b) and motifs (c). Exon/intron structures were obtained from the Gene Structure Display Server. Motifs were investigated using the Multiple Expectation Maximization for Motif Elucidation (MEME) system.

A diagram of exon/intron structure and motifs is shown in Figure 4. To make the results clearer, we first constructed a phylogenetic tree using only the *P. trichocarpa* protein sequences, which were grouped into three subgroups in accordance with the phylogenetic tree mentioned above (Figure 4(a)). From the exon/intron structure diagram in Figure 4(b), we found that almost all of the genes had twelve exons but in different distribution ranges. However, *PtrABC1P1* and *PtrABC1P2*, the members of group Ib, had roughly the same structure; both contained four exons that shared the same distribution. MEME motif detection revealed the diversification of the* Populus ABC1* genes. The details of the 17 putative motifs identified are shown in Table 2.  Figure 4(c) shows that genes in the same group share the same motifs and all of the genes contain motifs 1 and 7. This demonstrated the reliability of the phylogenetic classification.

**Figure 5:**
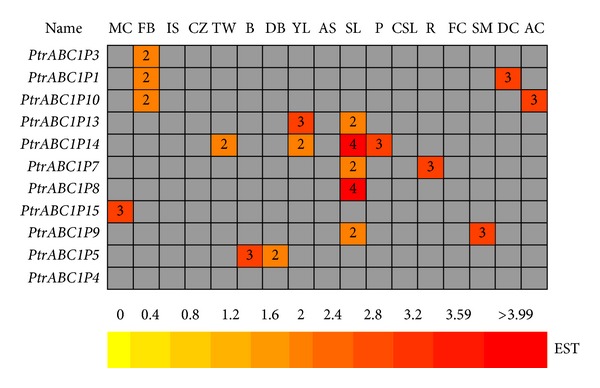
*In silico* EST analysis of *P. trichocarpa ABC1 *genes. The EST frequency for each gene was calculated by evaluating its EST representation among 17 cDNA libraries available at PopGenIE (http://www.popgenie.org/) [21]. The heatmap was visualized using the Heatmapper Plus tool by counting the corresponding ESTs for each particular gene in the database. The color bar at the bottom represents the frequencies of the EST counts. MC: male catkins, FB: flower buds, IS: imbibed seeds, CZ: cambial zone, TW: tension wood, B: bark, DB: dormant buds, YL: young leaves, AS: apical shoot, SL: senescing leaves, P: petioles, CSL: cold stressed leaves, R: roots, FC: female catkins, SM: shoot meristem, DC: dormant cambium, and AC: active cambium.

Publicly available Expressed Sequence Tags (ESTs) provided a useful tool to survey gene expression profiles in the form of a heatmap. We first carried out a preliminary analysis of *ABC1 *gene expression under various growth conditions and across different tissues by manual counting (Figure 5). Complete searching of the digital expression profiles from PopGenIE (http://www.popgenie.org/) yielded a total of 11 *Populus ABC1 *genes in the cDNA libraries. In the heatmap, most of the genes were represented by one or two ESTs in the cDNA libraries. *PtrABC1P14* and *PtrABC1P8 *had notably high expression in senescing leaves. These expression profiles demonstrated that most of the *ABC1* genes had a broad expression pattern across different tissues.

Promoter sequences (−2000 bp) were obtained from the NCBI database, and the *cis*-elements of these 17 genes were examined using the Plant Care database (Table 3). Many *cis*-elements were involved in abiotic stress responses such as the ABRE, MYB, MYC, and DRE core motifs and the LTRE motif. Most of the 17 genes contained ABRE and G-box motifs, both of which are related to the ABA signal transduction pathway. In addition, *PtrABC1P1*, *5*, *14*, and *15* also contained the W-box, which is found in the promoter regions of disease resistance-related genes and has been correlated with drought and ABA stress.

**Figure 6:**
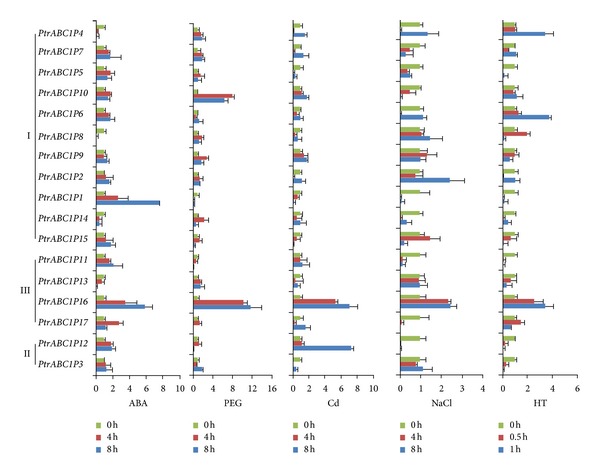
Relative expression of the 17 *P. trichocarpa ABC1* genes. The five treatments investigated were ABA, PEG, Cd, NaCl, and HT (high temperature). I, II, and III represent the three groups shown in the phylogenetic tree in Figure 2. 0 h represents no treatment. 0.5, 1, 4, and 8 h represent the corresponding times under different treatments.

To study the relative expression levels of the 17 *Populus ABC1* genes and select genes that had high relative expression levels, qRT-PCR was carried out using the SYBR Premix Ex Taq II kit.  Figure 6 shows the results. According to the phylogenetic tree, we grouped these genes into three groups, which are shown in the figure. Most of the genes had higher expression under ABA stress after 8 h treatment compared with 4 h, except* PtrABC1P17*. Among these genes, the relative expression levels of *PtrABC1P1* and *PtrABC1P16* were notably high, though *PtrABC1P1* was the highest. In the PEG treatment, we found that *PtrABC1P10 *and *PtrABC1P16* had the highest expression. Interestingly, five genes (*PtrABC1P8, 9*, *10*, *13*, and *14*) had higher expression after 4 h than after 8 h, while *PtrABC1P16* presented the opposite tendency. The expression levels of *PtrABC1P12 *and *PtrABC1P16* were high, with maximum relative expression values of 8, under Cd stress. The expression values were generally low under NaCl and high temperature (HT) stress. Only *PtrABC1P7* and *PtrABC1P13* showed high relative expression values (3 and 4, resp.) under NaCl treatment, while the expression values of *PtrABC1P4*, *6*, and *16* were about 3 under HT treatment.

## 4. Discussion

According to Lundquist et al. [15], there are 23 predicted *ABC1* genes in *P. trichocarpa*. However, the results of our modeling were a little different, and only 17 genes were found using the Pfam database. One of the reason for this might be that the other six genes are redundant or do not have ABC1 domains. In addition, in NCBI database, sequences of several genes were similar, and some of them were also incomplete. Taken together, only 17 genes were nonredundant and precise.

Previous data showed that the ABC1 proteins are involved in electron transfer in the respiratory chain and are located in mitochondria [6]. The first representative *ABC1* gene in plants, *AtABC1*, is a structural and functional homolog of yeast *ABC1*, and it allows partial restoration of the complex III activity of a yeast *abc1* mutant, suggesting the subcellular localization of *AtABC1* in mitochondria [4, 12]. However, *AtOSA1*, another *ABC1* gene in *Arabidopsis*, is located in chloroplasts and plays a role in the balance of oxidative stress [11]. In this study, most of the genes were located in mitochondria or chloroplasts. This further confirms that ABC1 family proteins contain the conserved ABC1 domain but have different kinase domains that lead to different localizations and presumably the diverse functions they perform. 

Construction of a phylogenetic tree by Lundquist et al. from the ABC1 kinase proteins of 42 diverse species of Archaea, bacteria, and eukaryotes revealed a division into 15 subfamilies, which were named ABC1 K1 to ABC1 K15 [15]. On the other hand, the phylogenetic tree was divided into three clear primary clades characterized by evolutionary origins and subcellular localization. The first clade comprised eight subfamilies (1–8) and was specific for photosynthetic organisms. The second clade consisted of subfamilies 11–15, which are all likely targeted to the mitochondria based on localization predictions. Among these families, subfamily 13 was singled out as it corresponds with ScCOQ8P. Experimental evidence supported the mitochondrial location assignment of subfamily 13, because ScCOQ8P is localized to the inner mitochondrial envelope [3], and the UQ pathway is localized within the mitochondria. The third, central clade with subfamilies 9 and 11 contained the majority of the nonphotosynthetic bacterial *ABC1Ps*. This taxonomy placed *PtrABC1 *in the first clade, which was specific for photosynthetic organisms. In another study, Yang and Li constructed a phylogenetic tree for rice and *Arabidopsis*, which was divided into three groups on the basis of orthologs or paralogs. In accordance with their study, we also constructed a three-group phylogenetic tree using MEGA5. We used the 17 *PtrABC1* genes in *P. trichocarpa* and 15 *ABC1* genes in rice together to create the tree. The genes in groups Ia and Ib were orthologs (*PtrABC1P1, 2, 4, 5, 6, 7, 8, 9, 10, 14, *and* 15*), *PtrABC1P3* and *12* were in group II, and group III contained *PtrABC1P11, 13, 16*, and *17*. 

Previous studies have revealed that the *Populus* genome has undergone at least three rounds of genome-wide duplication followed by multiple segmental duplication, tandem duplication, and transposition events such as retroposition and replicative transposition [30]. In particular, the segmental duplication associated with the salicoid duplication event that occurred 65 million years ago contributed remarkably to the expansion of many multigene families [31–36]. However, tandem duplication can easily occur in hotspots of chromosome recombination and has been closely correlated with the response of biotic and abiotic stress-related genes. In this study, about 82% (14 of 17) of the *Populus ABC1* genes were located in duplicated regions, while only three genes (*PtrABC1P5, 6, *and* 9*) were located outside of any duplicated blocks. Among these 14 genes, six were preferentially retained duplicates that were located in both duplicated regions, while the others were only on one of the blocks and lacked duplicates on the corresponding block, suggesting that dynamic rearrangement might have occurred that led to the loss of some genes and might explain why we only found 17 *PtrABC1* genes, while Lundquist et al. predicted 23. We draw attention to *PtrABC1P1* and *PtrABC1P2*, as they were in the same branch in the phylogenetic tree and were also found in homologous regions on chromosomes 4 and 11, respectively. This discovery might explain the homology of the two genes.

The heatmap showed that *PtrABC1P1*, *3*, and *10* had high expression in flower buds, with expression values of 2, probably indicating that these genes have a close relationship with flower differentiation and floral organ formation.* PtrABC1P14* had high expression in young leaves, senescing leaves, and petioles, suggesting another relationship between this gene and leaves. In contrast, *PtrABC1P4* had no expression in the 17  EST libraries. It might have expression in other libraries for which data was not available. For *PtrABC1P14*, it also highly expressed in TW (tension wood), which had a probability that this gene related to cellulose synthesis. Compared with herbaceous plant, *P. trichocarpa* had abundant wood as a woody plant. Wood is composed chiefly of cell walls, and the wall of a wood cell consists of compound middle lamella and secondary wall layers (S1, S2, and S3) composed of a cellulose/hemicellulose network impregnated with lignin. In many angiosperm trees, including *Populu*s, the normal development of secondary wall biosynthesis is greatly modified when tension wood is formed as a gravitational response to stem movements caused, for example, by wind or load [37–39]. One of the main characteristics of tension wood, which distinguishes it from normal wood, is the formation of fibers with a thick inner gelatinous cell wall layer mainly composed of crystalline cellulose. Hence, tension wood is enriched in cellulose and deficient in lignin and hemicelluloses [40, 41]. In view of these factors and high expression in TW, *PtrABC1P14 was speculated that it might* relate to the secondary cell wall formation and cellulose synthesis in *ABC1* gene family which might make a contribution to wood formation study.

Previous studies showed that *ABC1* genes function differently in different plants. In *S. cerevisiae*, an ABC1 protein was shown to have chaperone-like activity and be essential for the proper conformation of cytochrome *b* complex III [6]. However, the ABC1 proteins in prokaryotes described as a new family of putative kinases [2] might be involved in the regulation of UQ biosynthesis [7]. Moreover, the homolog of the yeast ABC1 protein in *Arabidopsis* can partially restore respiratory complex deficiency when expressed in *S. cerevisiae* [12]. The chloroplast AtOSA1 (AtABC1P17) protein was identified as a new factor playing a role in the balance of oxidative stress. There was also a study that showed that *ZmABC1-10* was significantly upregulated and stable under elevated Cd^2+^ concentrations [42]. Using transgenic plants, Wang et al. demonstrated that *TaABC1* overexpression enhanced drought, salt, and cold stress tolerance in *Arabidopsis* and suggested that *TaABC1* might act as a regulatory factor involved in multiple stress responses [19]. In this study, similar results were obtained. All 17 *PtrABC1* genes, except for *PtrABC1P17*, had ABRE or G-box *cis*-elements, which play an important role in drought and ABA tolerance. This result was supported by qRT-PCR analysis. The qRT-PCR results showed that many genes were highly expressed under ABA and PEG treatment, such as *PtrABC1P7*, *10*, *2*, *15*, *11*, and *16*. The expression of some genes under Cd stress also presented a similar tendency. Interestingly, *PtrABC1P16* was highly expressed in all five treatments. However, members of the same group were only expressed under a subset of the conditions; *PtrABC1P17* was only highly expressed under Cd stress, while *PtrABC1P11* and *13* showed high expression under ABA and Cd stress and PEG, Cd, NaCl, and HT stress, respectively. To summarize, the *ABC1Ps* in *Populus* were differentially expressed in response to heat, ABA, PEG, NaCl, and CdCl_2_ stress, which implies that the *PtrABC1Ps* play different but important roles in response to abiotic stresses. These results might help us to select highly expressed genes that can lay a foundation for subsequent studies on the *PtrABC1* genes in *Populus*.

## 5. Conclusions

 The results of this study provide a phylogenetic and bioinformatic overview of 17 *PtrABC1* genes in *P. trichocarpa*, most of which are localized to the mitochondria or chloroplasts. A phylogenetic tree showed the phylogeny and evolutionary relationships of the *PtrABC1 *genes, while heatmap analysis showed their expression levels in different tissues. Exon-intron structure and motif analyses were carried out to further investigate the phylogeny. *Cis*-elements contained in the *PtrABC1* genes provide clues to their functions and expression under different abiotic stresses. From this, we should be able to select genes related to stress responses for further studies. However, the specific functions of the *PtrABC1* genes remain unknown. Hence, further studies should be carried out to improve on the results of this study.

## Figures and Tables

**Table 1:** *ABC1* gene family in *P.  trichocarpa*.

Gene name^a^	Accession number^b^	NCBI Locus ID^c^	Length^d^	Localization^e^
* PtrABC1P1*	POPTR_0004s03280	XM_002305610.1	630	chlo
*PtrABC1P2 *	POPTR_0011s04110	XM_002316632.1	628	chlo
*PtrABC1P3 *	POPTR_0004s07340	XM_002305222.1	518	chlo
*PtrABC1P4 *	POPTR_0002s06180	XM_002300851.1	734	nucl
*PtrABC1P5 *	POPTR_0002s00200	XM_002301843.1	704	chlo
*PtrABC1P6 *	POPTR_0010s22780	XM_002316324.1	826	chlo
*PtrABC1P7 *	POPTR_0001s14410	XM_002297781.1	807	mito
*PtrABC1P8 *	POPTR_0006s29050	XM_002330776.1	737	chlo
*PtrABC1P9 *	POPTR_0019s08950	XM_002329202.1	543	mito
*PtrABC1P10 *	POPTR_0018s03080	XM_002324321.1	712	mito
*PtrABC1P11 *	POPTR_0012s09520	XM_002318076.1	471	cyto
*PtrABC1P12 *	POPTR_0008s06780	XM_002311182.1	538	mito
*PtrABC1P13 *	POPTR_0007s06450	XM_002310562.1	616	chlo
*PtrABC1P14 *	POPTR_0005s08490	XM_002307087.1	1235	chlo
*PtrABC1P15 *	POPTR_0014s16660	XM_002320491.1	719	cyto
*PtrABC1P16 *	POPTR_0002s18630	XM_002515113.1	616	chlo
*PtrABC1P17 *	POPTR_0018s04280	XM_002324089.1	242	nucl

^a^Systematic designation given to *P.  trichocarpa ABC1Ps*.

^b^Phytozome locus ID of full-length cDNA sequence available at Phytozome (http://www.phytozome.net/search.php).

^c^Accession number of NCBI locus ID(http://www.ncbi.nlm).

^d^Length of predicted gene product (amino acids).

^e^Localization of ABC1 proteins as predicted by WoLF PSORT (http://wolfpsort.org/).

**Table 2:** Motif sequences of the *P.  trichocarpa  ABC1* genes identified using MEME tools.

Motif	Width	Best possible match
1	41	VEEELGAPVDDIFDQFDYEPIAAASLGQVHRARLKGQEVVI
2	25	AVESYLEQILSHGFFHADPHPGNIA
3	29	EYVKVPAIYWEYTTPQVLTMZYVPGIKIN
4	29	PTFVKLGQGLSTRPDICPPEYLEELAELQ
5	50	IGEDLLSIAADQPFRFPATFTFVVRAFSVLDGIGKGLDPRFDITEIAKPY
6	29	IYDECASVLYQEIDYTKEAANAELFASNF
7	27	KVQRPGLKDLFDIDLKNLRVIAEYLQK
8	22	GRLIFYDFGMMGSISPNIREGL
9	33	YGIYEKDPDKVLEAMIQMGVLVPTGDMTAVRRT
10	50	YSTIQRTLEIWGSVLTFIFKAWLNNQKFSYRGGMTEEKKMVRRKALAKWL
11	50	RQSRAFHNLFRQADRVQKLAETIQRLEQGDLKLRVRTLEAERAFQRVAAV
12	48	HGTVVSVCLHLPQFRNYSQYSFPSREYSSFALCNIKEQFGRRCFTRNY
13	41	AIYLAILFSPSMMMAPFADSCGPEFRKIWLHVVHRTLEKAG
14	29	QNCPNPKAFIEEVEESFTFWGTPEGDLVH
15	11	DQVPPFPSETA

**Table 3:** *Cis*-elements of the 17 *ABC1* genes related to abiotic stresses such as drought and ABA. These *cis*-elements were obtained from the Plant Care database (http://bioinformatics.psb.ugent.be/webtools/plantcare/html/).

Gene name	*Cis*-elements related to abiotic stress
*PtrABC1P1 *	G-box; W box
*PtrABC1P2 *	ABRE; G-box
*PtrABC1P3 *	ABRE; G-box
*PtrABC1P4 *	ABRE
*PtrABC1P5 *	G-box; W box
*PtrABC1P6 *	ABRE; G-box
*PtrABC1P7 *	ABRE; G-box
*PtrABC1P8 *	ABRE; G-box
*PtrABC1P9 *	ABRE; G-box
*PtrABC1P10 *	ABRE; G-box
*PtrABC1P11 *	G-box
*PtrABC1P12 *	G-box
*PtrABC1P13 *	ABRE; G-box
*PtrABC1P14 *	ABRE; G-box; W box
*PtrABC1P15 *	ABRE; G-box; W box
*PtrABC1P16 *	ABRE; G-box
*PtrABC1P17 *	None

**Table 4:** Primers for the 17 *ABC1* genes and *Actin* used in qRT-PCR. F represents forward primer while R represents reverse primer. All primers are listed in the 5′-3′ direction.

Gene name	Primer (5′-3′)
*PtrABC1P1 *	F TTGAATTGGCTGAGACTGGATG R CACAGGCTTGGGAAAAGAAACA
*PtrABC1P2 *	F TTGAAGAGGTTCCTGTAGCATCTGG R GTCTAACCTTTACAGCAACCACCGT
*PtrABC1P3 *	F CGAGGTACATTTACGATCTGCGA R GCAACAAACTGTCCAGCTTTCAC
*PtrABC1P4 *	F TGACGGACAGCTCACCACAGTT R TGTTCTGGCACGAAGGGACTCT
*PtrABC1P5 *	F CGTAACTTCTTTGATGACGCACTC R AACAGCACCCAGACCATTCACTAG
*PtrABC1P6 *	F GGTGTTTACTGCATTTGCTACCG R TACCATCCCCAAATCGTACTGTG
*PtrABC1P7 *	F AAGGACCTTCCACAGGTTGTTG R GCTTTTCTCCCTCGATCCACTC
*PtrABC1P8 *	F CCGAAACAGTGAGGAGTAAAGTGC R CCAGATTAGACCAATACAGTCCCA
*PtrABC1P9 *	F TTCCAATTCCAAGCAGGAGATC R CCACAAGTTTGGGCAAGTTTTC
*PtrABC1P10 *	F TTCCATGCTGATCCTCATCCAG R TTGCCAAACCCAGAGAGTCACG
*PtrABC1P11 *	F GGACAAATGATACTGAAGAGCGG R CAAGCCTCAACTTATCTGGGAGA
*PtrABC1P12 *	F TCAGACAATGAGGGAGTCAATGCT R GAGCTACATGAACTTGTGCAAGGG
*PtrABC1P13 *	F TTGGGAATACACCACACCGC R AGGGTCGGCATGGAAGAATC
*PtrABC1P14 *	F AGAAAAAGAAGCAACGTCTGGC R CAACAAATGTGAAAGTGGCAGG
*PtrABC1P15 *	F GGATGAGCTTGCCAAGTTGC R GCTGGTCCTCAAACGCCTTA
*PtrABC1P16 *	F AGCAATAGTCTCCGTCACCGAT R ATTCTTTGGGCTTAGGAGGCGT
*PtrABC1P17 *	F CACAAGTCCATCGTGCGACATT R GGGCTGAAGTTATACTGCGGTT
*Actin *	F CATCAAAGCATCGGTGAGGTC R GTTGCCATCCAGGCTGTCC

## References

[1] Kannan N, Taylor SS, Zhai Y, Venter JC, Manning G (2007). Structural and functional diversity of the microbial kinome. *PLoS Biology*.

[2] Leonard CJ, Aravind L, Koonin EV (1998). Novel families of putative protein kinases in bacteria and archaea: evolution of the ‘eukaryotic’ protein kinase superfamily. *Genome Research*.

[3] Bousquet I, Dujardin G, Slonimski PP (1991). *ABC1*, a novel yeast nuclear gene has a dual function in mitochondria: it suppresses a cytochrome b mRNA translation defect and is essential for the electron transfer in the *bc1* complex. *EMBO Journal*.

[4] Do TQ, Hsu AY, Jonassen T, Lee PT, Clarke CF (2001). A defect in coenzyme Q biosynthesis is responsible for the respiratory deficiency in *Saccharomyces cerevisiae abc1* mutants. *Journal of Biological Chemistry*.

[5] Brasseur G, Tron P, Dujardin G, Slonimski PP, Brivet-Chevillotte P (1997). The nuclear *ABC1* gene is essential for the correct conformation and functioning of the cytochrome *bc1* complex and the neighbouring complexes II and IV in the mitochondrial respiratory chain. *European Journal of Biochemistry*.

[6] Hsieh EJ, Dinoso JB, Clarke CF (2004). A tRNA^TRP^ gene mediates the suppression of *cbs2-223* previously attributed to *ABC1/COQ8*. *Biochemical and Biophysical Research Communications*.

[7] Vidi P-A, Kanwischer M, Baginsky S (2006). Tocopherol cyclase (VTE1) localization and vitamin E accumulation in chloroplast plastoglobule lipoprotein particles. *Journal of Biological Chemistry*.

[8] Ytterberg AJ, Peltier J-B, Van Wijk KJ (2006). Protein profiling of plastoglobules in chloroplasts and chromoplasts. A surprising site for differential accumulation of metabolic enzymes. *Plant Physiology*.

[9] Lundquist PK, Poliakov A, Bhuiyan NH, Zybailov B, Sun Q, van Wijk KJ (2012). The functional network of the *Arabidopsis* plastoglobule proteome based on quantitative proteomics and genome-wide coexpression analysis. *Plant Physiology*.

[10] Jasinski M, Sudre D, Schansker G (2008). AtOSA1, a member of the *ABC1*-like family, as a new factor in cadmium and oxidative stress response. *Plant Physiology*.

[11] Xie LX, Hsieh EJ, Watanabe S (2011). Expression of the human atypical kinase ADCK3 rescues coenzyme Q biosynthesis and phosphorylation of Coq polypeptides in yeast *coq8* mutants. *Biochimica et Biophysica Acta*.

[12] Cardazzo B, Hamel P, Sakamoto W, Wintz H, Dujardin G (1998). Isolation of an *Arabidopsis thaliana* cDNA by complementation of a yeast *ABC1* deletion mutant deficient in complex III respiratory activity. *Gene*.

[13] Majeran W, Friso G, Asakura Y (2012). Nucleoid-enriched proteomes in developing plastids and chloroplasts from maize leaves: a new conceptual framework for nucleoid functions. *Plant Physiology*.

[14] Yang SG, Li T, Liu M (2012). Phylogenetic, structure and expression analysis of *ABC1Ps* gene family in rice. *Biologia Plantarum*.

[15] Lundquist PK, Davis JI, van Wijk KJ (2012). ABC1K atypical kinases in plants: filling the organellar kinase void. *Trends in Plant Science*.

[16] Hanks SK, Hunter T (1995). The eukaryotic protein kinase superfamily: kinase (catalytic) domain structure and classification. *FASEB Journal*.

[17] Taylor SS, Radzio-Andzelm E (1994). Three protein kinase structures define a common motif. *Structure*.

[18] Dissmeyer N, Schnittger A (2011). The age of protein kinases. *Methods in Molecular Biology*.

[19] Wang C, Jing R, Mao X, Chang X, Li A (2011). *TaABC1*, a member of the activity of *bc1*complex protein kinase family from common wheat, confers enhanced tolerance to abiotic stresses in *Arabidopsis*. *Journal of Experimental Botany*.

[20] Douglas CJ, DiFazio SP, Jansson S, Bhalerao R, Groover A (2010). The *Populus* genome and comparative genomics. *Genetics and Genomics of Populus*.

[21] Sjödin A, Street NR, Sandberg G, Gustafsson P, Jansson S (2009). The *Populus* Genome Integrative Explorer (PopGenIE): a new resource for exploring the *Populus* genome. *New Phytologist*.

[22] Horton P, Park K-J, Obayashi T (2007). WoLF PSORT: protein localization predictor. *Nucleic Acids Research*.

[23] Thompson JD, Gibson TJ, Plewniak F, Jeanmougin F, Higgins DG (1997). The CLUSTAL X windows interface: flexible strategies for multiple sequence alignment aided by quality analysis tools. *Nucleic Acids Research*.

[24] Hall TA (1999). BioEdit: a user-friendly biological sequence alignment editor and analysis program for Windows 95/98/NT. *Nucleic Acids Symposium Series*.

[25] Saitou N, Nei M (1987). The neighbor-joining method: a new method for reconstructing phylogenetic trees. *Molecular Biology and Evolution*.

[26] Tamura K, Peterson D, Peterson N, Stecher G, Nei M, Kumar S (2011). MEGA5: molecular evolutionary genetics analysis using maximum likelihood, evolutionary distance, and maximum parsimony methods. *Molecular Biology and Evolution*.

[27] Segerman B, Jansson S, Karlsson J (2007). Characterization of genes with tissue-specific differential expression patterns in *Populus*. *Tree Genetics and Genomes*.

[28] Sterky F, Bhalerao RR, Unneberg P (2004). A *Populus* EST resource for plant functional genomics. *Proceedings of the National Academy of Sciences of the United States of America*.

[29] Zhang X, Wollenweber B, Jiang D, Liu F, Zhao J (2008). Water deficits and heat shock effects on photosynthesis of a transgenic *Arabidopsis thaliana* constitutively expressing *ABP9*, a bZIP transcription factor. *Journal of Experimental Botany*.

[30] Tuskan GA, DiFazio S, Jansson S (2006). The genome of black cottonwood, *Populus trichocarpa* (Torr. & Gray). *Science*.

[31] Wilkins O, Nahal H, Foong J, Provart NJ, Campbell MM (2009). Expansion and diversification of the *Populus* R2R3-MYB family of transcription factors. *Plant Physiology*.

[32] Barakat A, Bagniewska-Zadworna A, Choi A (2009). The cinnamyl alcohol dehydrogenase gene family in *Populus*: phylogeny, organization, and expression. *BMC Plant Biology*.

[33] Kalluri UC, Difazio SP, Brunner AM, Tuskan GA (2007). Genome-wide analysis of *Aux/IAA* and *ARF* gene families in *Populus trichocarpa*. *BMC Plant Biology*.

[34] Hu R, Qi G, Kong Y, Kong D, Gao Q, Zhou G (2010). Comprehensive analysis of NAC domain transcription factor gene family in *Populus trichocarpa*. *BMC Plant Biology*.

[35] Barakat A, Choi A, Yassin NBM, Park JS, Sun Z, Carlson JE (2011). Comparative genomics and evolutionary analyses of the *O*-methyltransferase gene family in *Populus*. *Gene*.

[36] Zhuang J, Cai B, Peng R-H (2008). Genome-wide analysis of the *AP2/ERF* gene family in *Populus trichocarpa*. *Biochemical and Biophysical Research Communications*.

[37] Hellgren JM, Olofsson K, Sundberg B (2004). Patterns of auxin distribution during gravitational induction of reaction wood in poplar and pine. *Plant Physiology*.

[38] Mellerowicz EJ, Baucher M, Sundberg B, Boerjan W (2001). Unravelling cell wall formation in the woody dicot stem. *Plant Molecular Biology*.

[39] Pilate G, Déjardin A, Laurans F, Leplé J-C (2004). Tension wood as a model for functional genomics of wood formation. *New Phytologist*.

[40] Fujita M, Saiki H, Harada H (1974). Electron microscopy of microtubules and cellulose microfibrils in secondary wall formation of popular tension wood fibers. *Mokuzai Gakkaishi*.

[41] Norberg PH, Meier H (1966). Physical and chemical properties of the gelatinous layer in tension wood fibres of aspen (*Populus tremula* L.). *Holzforschung*.

[42] Gao Q-S, Yang Z-F, Zhou Y (2010). Cloning of an *ABC1*-like gene *ZmABC1-10* and its responses to cadmium and other abiotic stresses in maize (*Zea mays* L.). *Acta Agronomica Sinica*.

